# Tumor–Stroma Ratio in Head and Neck Squamous Cell Carcinoma: A Systematic Review and Meta‐Analysis

**DOI:** 10.1111/odi.15319

**Published:** 2025-03-24

**Authors:** Gennaro Musella, Martina Coppini, Fábio França Vieira E Silva, Giuseppina Campisi, Mario Pérez‐Sayáns, Vito Carlo Alberto Caponio, Alejandro I. Lorenzo‐Pouso

**Affiliations:** ^1^ Department of Clinical and Experimental Medicine University of Foggia Foggia Italy; ^2^ Department of Precision Medicine in Medical, Surgical and Critical Care University of Palermo Palermo Italy; ^3^ Oral Medicine, Oral Surgery and Implantology Unit (MedOralRes Group), Faculty of Medicine and Dentistry Universidade de Santiago de Compostela A Coruña Spain; ^4^ Health Research Institute of Santiago de Compostela (IDIS) Santiago de Compostela Spain; ^5^ Department of Biomedicine, Neuroscience and Advanced Diagnostics University of Palermo Palermo Italy; ^6^ Institute of Materials of Santiago de Compostela (iMATUS) A Coruña Spain

**Keywords:** biomarker, head and neck cancer, head and neck neoplasms, prognostic, systematic review and meta‐analysis, tumor–stroma, tumor–stroma ratio

## Abstract

**Background:**

Tumor‐stroma ratio (TSR) has been recognized as a significant prognostic factor in various cancers. This systematic review evaluates the role of TSR in head and neck squamous cell carcinoma (HNSCC) and its association with patient outcomes such as overall survival (OS), disease‐free survival (DFS), disease‐specific survival (DSS), and lymph node metastasis (LNM).

**Methods:**

A comprehensive search was conducted across Scopus, Embase, PubMed, and Web of Science. Nineteen studies were included. Data extraction and quality assessment were performed progressively, with meta‐analyses. All studies assessed TSR using hematoxylin and eosin staining of tissue samples. The meta‐analyses focused on the impact of TSR on OS, DFS, DSS, and LNM, providing pooled hazard ratios (HRs) and odds ratios (ORs) with corresponding confidence intervals (CIs).

**Results:**

Meta‐analysis revealed a significant association between TSR and OS (HR 1.99, 95% CI 1.71–2.32, *p* < 0.001), DFS (HR 2.07, 95% CI 1.80–2.39, *p* < 0.001), DSS (HR 2.33, 95% CI 1.95–2.78, *p* < 0.001), and LNM (OR 1.76, 95% CI 1.15–2.70, *p* = 0.01). Minimal to low heterogeneity was detected among studies, and no publication bias was observed.

**Conclusions:**

TSR can effectively identify high‐risk patients, being a reliable prognostic marker that could be readily integrated into routine pathology practice for HNSCC.

## 1 Introduction

Head and neck squamous cell carcinoma (HNSCC), encompassing epithelial tumors originating from the mucosa of the oral cavity, oropharynx, hypopharynx, and larynx, is among the most prevalent cancers globally. Annually, nearly 650,000 new cases are diagnosed worldwide, resulting in over 300,000 cancer‐related deaths (Bray et al. 2024). Despite standard‐of‐care therapies, including surgery, radiation, and/or platinum‐ or taxane‐based chemotherapy, the 5‐year survival rate for HNSCC remains around 50% due to treatment resistance (Perrotti et al. 2022; Caponio et al. 2024).

The therapeutic approach and prognostic evaluation for HNSCC heavily rely on disease staging, which may not fully capture the diverse biological behaviors of these tumors (Farah 2021; Caponio et al. 2020). Even in early stages, aggressiveness can be pronounced, leading to increased invasion, metastasis, and a poor prognosis (Kumar et al. 2022). Numerous biomarkers have been proposed as prognostic indicators for HNSCC (Nocini et al. 2022), with specific histopathological features being prominent among them (Dolens et al. 2021; Silva et al. 2024; Caponio et al. 2023). Indeed, in the last update of the American Joint Committee on Cancer (AJCC) TNM staging system, the introduction of the depth of invasion and the extranodal extension partially improved the stratifying ability of the TNM staging system in the prognostication of these patients (Mascitti et al. 2018). These microscopic characteristics, either individually or combined into grading systems, may represent potential biomarkers to differentiate patients with HNSCC. Numerous histopathological characteristics, for example, perineural invasion (Caponio et al. 2023), tumor‐associated eosinophilia (Caponio et al. 2022) among others, have been investigated for their prognostic significance (Dolens et al. 2021). Recently, the tumor‐stroma ratio (TSR) has emerged as a novel parameter of interest in HNSCC prognostics (Almangush et al. 2018; Mascitti et al. 2020). While stroma in healthy tissues typically impedes cancer spread, its presence within the tumor tissue can paradoxically facilitate its progression (Mueller and Fusenig 2004). TSR, defined as the proportion of tumor tissue relative to the surrounding stromal tissue at the invasive front, has been recognized as a valuable prognostic feature across various solid tumors. Initially proposed by Mesker et al. for colorectal cancer, TSR assessment on H&E‐stained sections has now extended to other cancer types (Mesker et al. 2007), with growing evidence applied to HNSCC. To date, although three systematic reviews have explored the impact of TSR on prognosis and clinicopathological variables in HNSCC, they have faced limitations (Dolens et al. 2021; Almangush et al. 2021a; Morais et al. 2022). One review failed to conduct a pooled analysis (Morais et al. 2022), while others did not perform subgroup analyses or consider relevant secondary outcomes (Dolens et al. 2021; Almangush et al. 2021a). Moreover, new original investigations have been published on this topic. These deficiencies have limited a comprehensive understanding of TSR influence on HNSCC outcomes and its interaction with specific clinical and pathological factors. Building on this background, this study proposes a systematic review and meta‐analysis to investigate the impact of TSR characteristics on the prognosis and clinicopathological features of HNSCC, complemented by trial sequential analysis (TSA) for time‐to‐event outcomes to assess the statistical power of the findings.

## 2 Materials and Methods

The protocol of this meta‐analysis was registered in The International Database of Prospectively Registered Systematic Reviews (PROSPERO), protocol number CRD42024531301, and was reported following the Preferred Reporting Items for Systematic Reviews and Meta‐analyses (PRISMA) guidelines (Page et al. 2021).

### 2.1 Search Strategy and Database Screening

The search strategy included querying PubMed, Embase, Web of Science, and Scopus for all relevant studies from inception until July 2024. Thesaurus terms (MeSH and EMTREE) for each database were combined with free terms to enhance search comprehensiveness. The general search strategy in PubMed was:

((“Head and Neck Neoplasm”[MeSH Terms]) OR “Head and Neck Cancer”[Title/Abstract] OR “Head and Neck Tumour”[Title/Abstract] OR “Oropharyngeal Cancer”[Title/Abstract] OR “Oropharyngeal Neoplasm”[Title/Abstract] OR “Oropharyngeal Tumor”[Title/Abstract] OR “Laryngeal Cancer”[Title/Abstract] OR “Laryngeal Neoplasm”[Title/Abstract] OR “Laryngeal Tumor”[Title/Abstract] OR “Laryngeal Tumor”[Title/Abstract] OR “Oral Cavity Cancer”[Title/Abstract] OR “Oral Cavity Neoplasm”[Title/Abstract] OR “Oral Cavity Tumor”[Title/Abstract] OR “Oral Cavity Tumour”[Title/Abstract] OR “Mouth Neoplasms”[Title/Abstract] OR “Squamous Cell Carcinoma”[Title/Abstract] OR “Carcinoma, Squamous Cell”[Title/Abstract] OR “Epidermoid Carcinomas”[Title/Abstract] OR “Planocellular Carcinoma”[Title/Abstract]) AND (“Histopathology”[MeSH Terms] OR “Tumor Grading”[Title/Abstract] OR “Tumor‐Stroma Ratio”[Title/Abstract] OR “Carcinoma–Stroma Ratio”[Title/Abstract] OR “Cancer/Carcinoma Percentage”[Title/Abstract]). Detailed search strategies for all databases can be accessed in Supporting Informations as Table S1. The search was not confined to specific countries or languages, and reference lists from relevant articles were manually examined to complement the search. Duplicate articles were removed automatically after import into EndNote reference software (Endnote X9.3.2, Clarivate Analytics).

### 2.2 Eligibility Criteria

#### 2.2.1 Inclusion Criteria

The inclusion criteria were as follows: (a) Prospective and retrospective clinical cohort studies focusing on the histopathologic assessment of TSR in samples from HNSCC patients; (b) Each study must include at least 20 patients; (c) Studies must analyze prognosis by calculating the hazard ratio (HR) and its 95% confidence interval (95% CI) for at least one of the following primary outcomes: overall survival (OS), disease‐free survival (DFS), or disease‐specific survival (DSS). Additionally, relevant secondary outcomes, prioritized for pooled analysis and expressed as odds ratios (ORs), include sex, staging, T status, lymph node metastasis (LNM) and tumor grade. When HRs or ORs were determined in both univariable and multivariable models, data were extracted from the multivariable model, which usually reflects a greater adjustment for potentially confounding factors. The authors of published studies were contacted for clarification or additional data requests. Whether the HR and 95% CI were not reported, but the relative Kaplan–Meier curve was originally included in the manuscript, the required information was retrieved according to the methods of Tierney et al. (2007).

#### 2.2.2 Exclusion Criteria

Systematic reviews and meta‐analyses, case reports, and case series were excluded. Cohort studies with missing information from which HRs or ORs could not have been retrieved, were also excluded. The same criterion was applied to studies involving animal models or patients in which TSR was assessed post chemo‐ or radiotherapy.

### 2.3 Focused PICO Question

In patients with HNSCC who underwent primary tumor resection or biopsy, what is the impact of high–low tumor–stroma ratio on OS, DFS, and DSS? Additionally, what is the relationship between tumor‐stroma ratio status and secondary outcomes such as sex, tumor staging, T status, LNM, and tumor grading?

Population: Patients diagnosed with HNSCC (ICD‐10 codes: C00‐C06, C09, C10, C11, C13, C14, and C32), who have undergone primary tumor resection or biopsy prior to any chemotherapy or radiation therapy.

Intervention: Histopathological assessment of TSR, indicating patients with high TSR.

Comparison: Histopathological assessment of TSR, indicating patients with low TSR.

Outcome: To determine the association between the histopathological assessment of TSR with the following primary outcomes: OS, DFS, DSS. Additionally, the relationship between TSR and some secondary outcomes, namely, sex distribution, tumor staging, T status, LNM, and tumor grading.

### 2.4 Study Screening and Inclusion

The titles and abstracts of references found during the search process were initially independently screened by two investigators (AILP and GM) to exclude clearly irrelevant studies. Full texts of relevant articles were obtained to determine whether the studies were eligible. At this stage, the k‐agreement calculation was evaluated to rank the reviewers' agreement. A third investigator (VCAC) was consulted to resolve disagreements on eligibility and categorization of studies.

### 2.5 Data Extraction

Two reviewers (GM and FFVS) independently completed an extraction sheet, which was at the end approved during a joint meeting. When there was a disagreement, a third author (AILP) helped reevaluate the articles. The extraction sheet featured several fields formatted in Excel that were captured: 1. first author's surname; 2. country of population enrollment, subsequently clustered into belonging continents; 3. year of publication; 4. type of outcome (OS, DSS, or DFS); 5. cancer subsite; 6. total sample size; 7. sample size divided into high‐ and low TSR; 7. magnification level; 8. staining; 9. follow‐up time; 10. staging edition; 11. cut‐offs to separate high‐ and low TSR; 12. type of statistical analysis (univariate/multivariate); 13. patients with high‐ and low TSR and LNM; 14. or high‐grade tumors (G2/G3); 15. or high T status of TNM staging (T3/T4); 16. or high TNM staging tumors (III/IV); and 17. the assessment in male and female patients.

### 2.6 Assessment of Risk of bias

Assessment of risk of bias was undertaken by using a customized criteria adapted from the Reporting Recommendations for Tumor Marker Prognostic Studies (REMARK) standards (Altman et al. 2012). Six crucial parameters were assessed as part of this evaluation: 1. sample size; 2. clinical data from the cohort; 3. histopathology/immunohistochemistry; 4. prognosis; 5. statistical methods; and 6. conventional prognostic factors. All parameters were rated as adequate (A), inadequate (I), or not available (NA), with an increase in the study's total quality score for each “adequate” grade. Every acceptable item raised each study's overall quality score. Table S2 shows the specific thresholds determined for each item based on previous reports (Altman et al. 2012). Two review participants (FFVS and MC) carried out this procedure independently, and disagreements were resolved in a combined meeting with a third author (GC).

### 2.7 Evaluation of Reliability Methodologies

The methodologies employed to ensure reliability in TSR assessment were analyzed across the included studies. When reported, inter‐observer and intra‐observer reliability values, such as Cohen's kappa, were also evaluated and reported in Table 1. However, a meta‐analysis of these reliability metrics could not be conducted due to the lack of data regarding percent agreement (*p*_o_) and chance agreement (*p*) (Sun 2011).

**TABLE 1:** Main characteristics and quality scores of included studies.

Authors	Remarks	Country	Year	Survival analysis	Subsite	Sample size	Cutoff	Reliability (κ)^a^
Alessandrini et al.	3	Italy	2024	DFS	Larynx	43	50%	NP
Almangush et al. (A)	4	Finland, Brazil	2018	DFS, DSS	Tongue	311	50%	0.91
Almangush et al. (B)	5	Brazil	2021	DFS, DSS, OS	Tongue	308	50%	NP
Almangush et al. (C)	5	Finland	2023	OS, DSS	Nasopharynx	115	50%	NP
Chang et al.	6	Taiwan	2024	OS, DSS	Oral cavity	162	50%	NP
Dourado et al.	6	Brazil	2020	DFS, DSS	Oral cavity	254	50%	0.96^b^
Huang et al.	6	China	2022	DFS, OS	Oral cavity	151	50%	0.906; 0.919, 0.946^c^
Kang et al.	6	China	2021	DFS, DSS	Tongue	103	50%	NP
Karpathiou et al.	4	French	2019	OS	Head & Neck	266	50%	NP
Knief et al.	5	Germany	2024	OS, DFS	Oral cavity	107	50%	NP
Mascitti et al.	5	Italy	2020	DFS, DSS, OS	Tongue	211	50%	0.807
Niranjan et al.	2	India	2018	DFS, OS	Oral cavity	60	50%	0.932
Qiu, Jiang & Shang	3	China	2022	DFS, DSS	Oral cavity	581	50%	NP
Sakai et al.	1	Japan	2022	DFS, LNM	Tongue	70	50%	NP
Silva et al.	3	Brazil	2023	DFS, DSS	Oral cavity	95	50%	NP
Sung et al.	6	South Corea	2020	DFS, OS	Oral cavity	256	Median [0.96 (0.53, 1.84)]	NP
Unlu et al.	4	Turkey	2013	OS	Larynx	85	50%	NP
Wang et al.	2	China	2023	OS, DFS, MFS	Oral cavity	114	50%	NP
Zhang et al.	4	China	2014	OS, DFS	Nasopharynx	93	50%	0.85

Abbreviations: DFS, disease‐free survival; DSS, disease‐specific survival; LNM, lymph node metastasis; NP, not present; OS, overall survival.

^a^ Reliability of TSR measurements (Cohen's kappa).

^b^ In this study, this value refers to intra‐rater reliability, as the assessment was conducted by a single operator.

^c^ In this study, both inter‐rater and intra‐rater reliability were calculated, with the first value reported for inter‐rater reliability and the second and third values for intra‐rater reliability of operator A and operator B, respectively.

### 2.8 Statistical Analysis for Survival Outcomes

HR and associated 95% CI for OS, DFS, and DSS outcomes were collected. Forest plots were used to illustrate pooled effect sizes. Moderators were defined as those variables which have might influenced the pooled outcome results. Moderators were “country”, “publication year”, “staging”, whether the included cohort was limited to early or advanced cancer stages, “magnification” for assessing the tumor‐stroma ratio, “subsite,” and “risk of bias”. Meta‐analyses for each outcome (OS, DFS and DSS) were carried out using either a fixed‐ or random‐effects model, depending on the level of heterogeneity identified. Heterogeneity was assessed using Cochran's *Q* test and *I* ^2^ index, where a cut‐off value of 50% distinguished low from high heterogeneity. A fixed‐effects model was adopted for low heterogeneity, and a random‐effects model was used for high heterogeneity (Higgins and Thompson 2002). Sensitivity analyses were conducted using the leave‐one‐out method to determine the impact of individual studies on the overall results (Willis and Riley 2017). Additional sensitivity analyses calculated the pooled effect sizes for each moderator, with statistical differences between groups assessed using the ANOVA Q‐test. Finally, potential publication bias was examined visually with funnel plots and statistically through trim and fill analysis and Egger's test.

### 2.9 Statistical Analysis for Clinical–Pathological Variables

Association among clinical‐pathological variables and TSR was estimated by pooling the total number of patients with rich (control group) and poor (experimental group) tumor stroma, focusing on outcomes such as LNM, G2/G3, stage III/IV, T stage 3/4, and sex. The Mantel–Haenszel method was used to pool the data in order to provide a cumulative OR and associated 95% CI (Review Manager software version 5.2.8; Cochrane Collaboration, Copenhagen, Denmark; 2014).

### 2.10 Trial Sequential Analysis

Trial sequence analysis (TSA), a cumulative meta‐analysis method, was employed to assess whether the number of trials and participants in a meta‐analysis was adequate to draw any conclusions (De Cassai et al. 2021) or if more research would be required. The data were analyzed using the ‘metacumbounds’ command from Stata Statistical Software version 14.0 (StataCorp, College Station, TX), as referenced in the study (Branko Miladinovic and Djulbegovic 2013). A fixed‐effect model was employed to determine each cumulative *z*‐value due to the low heterogeneity observed in the meta‐analysis (*I* ^2^ < 50%). The information size calculation was based on a priori anticipated intervention effect (APIS), assuming a 15% relative risk reduction (RRR) and 68.5% as the average survival rate (Barsouk et al. 2023) with 5% type I error and 20% type II error. We considered only DSS and DFS as outcomes of interest for TSA.

## 3 Results

### 3.1 Study Selection

**FIGURE 1:**
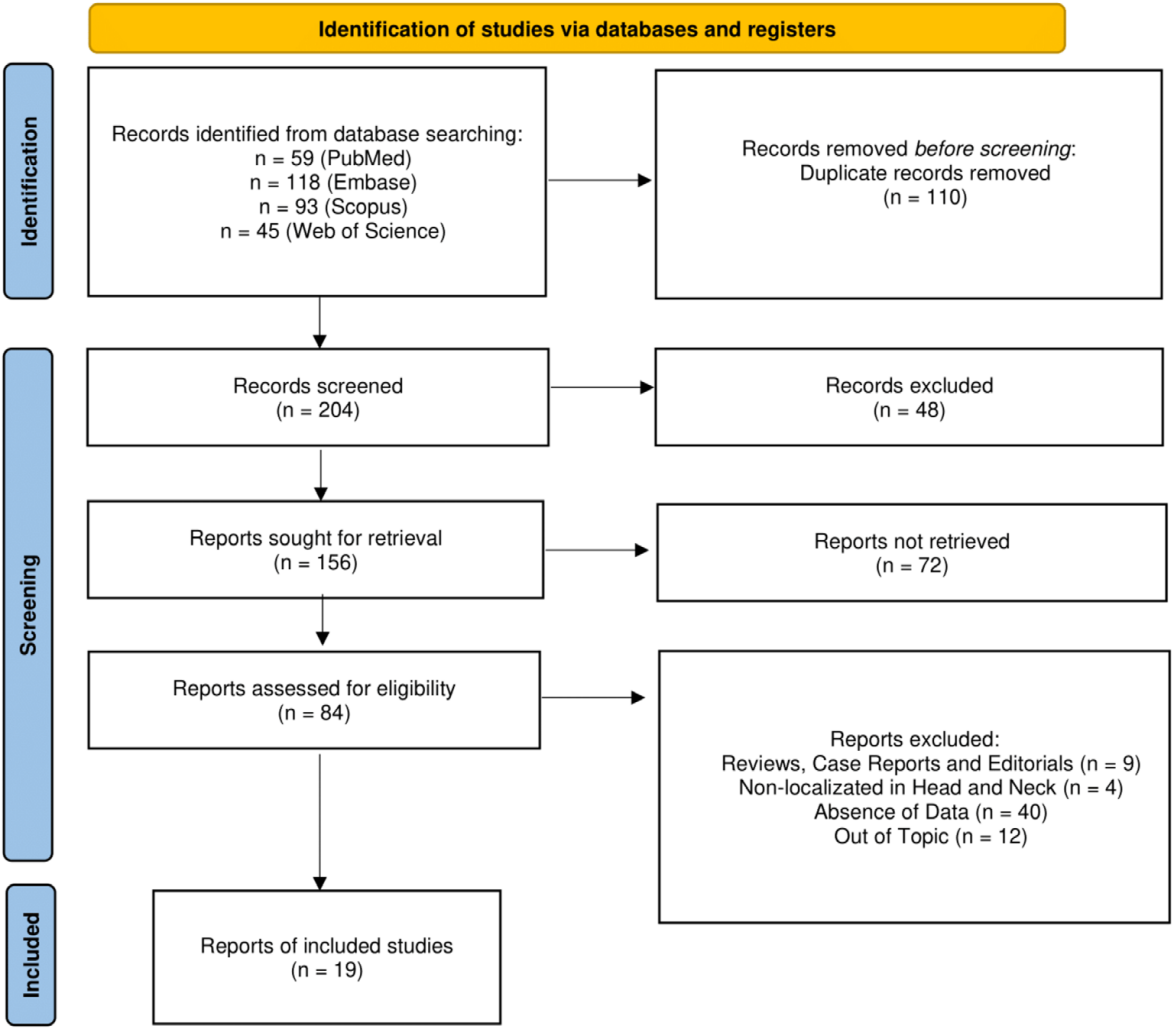
Flow diagram of study selection.

After removing the duplicates, our syntax resulted in 204 reports, from which 84 were included for full‐text assessment. Then, 65 did not meet the pre‐defined inclusion criteria and were removed. Finally, 19 were included in the systematic review for both qualitative evaluation and quantitative synthesis. The reviewers' agreement by k‐agreement score was 0.93. Figure 1 depicts a flow diagram representing comprehensively the study selection process.

### 3.2 Study Characteristics

A total of 3524 patients affected by HNSCC were included in the meta‐analysis. The population sample ranged between 43 and 581 cases, while the year of publication ranged from 2013 to 2024. Studies were conducted among 15 different countries on 3 continents: America, Asia, and Europe. Table S3 provides an in‐depth description of the included studies, whereas Table 1 contains a comprehensive summary description of their main characteristics.

### 3.3 Quality Assessment

Based on a cut‐off point > 4, 57.89% (11/19) of the studies were considered at low risk of bias (Almangush et al. 2021b, 2023; Chang and Lee 2024; Dourado et al. 2020; Huang et al. 2022; Kang et al. 2023; Knief et al. 2024; Mascitti et al. 2020; Silva et al. 2023; Zhang et al. 2014, 2020). Considering the maximum score of 6 as the standard, only 31.58% (6/19) of the studies were classified as adequate in all evaluated parameters (Table S4). In relation to the sample size domain of the 19 included studies, 4 (21.05%) were considered inadequate. Regarding the clinical data from the cohort, 9 (47.37%) reports were considered inadequate. In terms of histopathology or immunochemistry analysis, among the 19 included studies, only 2 (10.52%) were considered inadequate. According to the prognosis evaluation, 5 (26.31%) reports were considered inadequate. Statistical analysis varied widely across the 19 studies included; specifically, 6 (31.58%) reports were considered inadequate. Finally, regarding the inclusion of the conventional prognostic factors, 15.79% of reports were considered inadequate.

### 3.4 TSR Assessment Reliability Method

A certain degree of variability was observed in the methodologies used to ensure inter‐rater reliability in TSR assessment across the included studies.

Seven studies described evaluations conducted by two independent pathologists, with disagreements resolved through review sessions to reach consensus (Sakai et al. 2022; Kang et al. 2023; Alessandrini et al. 2022; Zhang et al. 2014; Almangush et al. 2024, 2021b, 2018).

A different approach was employed in one study, where a single operator assessed intra‐rater reliability by re‐evaluating the same sections after a 4‐weeks interval on a small sample (Dourado et al. 2020). Another investigation ensured reliability by having two researchers independently evaluate each slide twice to test inter‐observer reproducibility, resolving disagreements by involving a third reviewer who provided a final decision (Huang et al. 2022).

In one case, two pathologists independently evaluated histological sections, jointly identified the area to assess, and calculated the final score as the mean of their evaluations (Mascitti et al. 2020). By contrast, histological sections were simultaneously examined by two pathologists, with the final value determined as the unanimous evaluation of each parameter (Karpathiou et al. 2019).

Another study employed TSR estimation by two investigators, using the highest stromal percentage in case of disagreement (Niranjan and Sarathy 2018).

A combined manual and digital approach was utilized in one study, where the manual evaluation was performed by one pathologist and reviewed by another (Sung et al. 2021). Similarly, a mixed methodology included two observers recording slides three times to minimize intra‐observer variability, with disagreements resolved by a third observer (Qiu et al. 2023).

Two additional investigations followed similar methodologies but lacked precise details on how reliability was ensured during the manual evaluation phase (Wang et al. 2023; Unlu et al. 2013). Lastly, three studies provided no information about the number of pathologists involved or the methods used to address inconsistencies (Silva et al. 2023; Knief et al. 2024; Chang and Lee 2024).

Focusing on intra‐rater reliability, only three studies addressed this issue. One study involved a single operator who reassessed the same sections after a 4‐weeks interval to evaluate reliability (Dourado et al. 2020), while the other two studies involved repeated evaluations by two independent operators (Qiu et al. 2023; Wang et al. 2023).

### 3.5 Meta‐Analysis and Trial Sequential Analysis

The HRs are expressed as a measure of comparison between low TSR versus high TSR. A HR of 2.00 should be interpreted as a patient with a low TSR being 2 times more likely to undergo a negative outcome.

#### 3.5.1 Overall Survival

Meta‐analysis of the fixed‐effects model for OS showed an HR of 1.99 (1.71–2.32; *p*‐value < 0.001) (Figure S1A). Heterogeneity was low between studies (*I* ^2^ = 37.52%). Considering subgroup analysis, when discussing the differences between tumor subsites, the random‐effects model demonstrated an HR of 2.37 (1.56–3.60, *I* ^2^ = 53.16%) in the oral cavity, and the fixed‐effects model showed an HR of 2.11 (1.37–3.23, *I* ^2^ = 0%) in the nasopharynx and an HR of 1.48 (1.11–1.97, *I* ^2^ = 0%) in the tongue. There was only one study conducted on the larynx (Table S5) (ANOVA‐Q test *p*‐value = 0.165). According to the staging, fixed‐effects model HR was 2.14 (1.80–2.55, *I* ^2^ = 0%) for studies including any cancer stage, and random‐effects model HR was 2.83 (0.58–13.81, *I* ^2^ = 83.35%) for studies considering cT1/2‐N0 stage tumors (Table S5) (ANOVA‐Q test *p*‐value = 0.054). As for the different continents, the fixed‐effects model showed an HR of 2.39 (1.83–3.13) in Asia with low to moderate heterogeneity (*I* ^2^ = 44.95%) and an HR of 1.82 (1.51–2.19) in Europe with a very low heterogeneity (*I* ^2^ = 14.67%) (Table S5) (ANOVA‐Q test *p*‐value = 0.103). Meta‐regression by year of publication found no statistically significant correlation (*p*‐value of the fixed‐effects model = 0.829), concluding that the effect size did not depend on the year of publication (Figure S4). Leave‐one‐out demonstrated a strong consistency in our results (Figure S5). Publication bias was represented by the trim and fill method, which found 1 new unpublished “study” through the fixed‐effects model, fully illustrated by the funnel plot. On the other hand, Egger's linear regression test showed the absence of publication bias (pEgger = 0.404) with an estimated HR of 1.95 (1.67–2.27; *p*‐value < 0.001) (Figure S1B). Regarding RoB assessment, meta‐regression showed no statistically significant difference (*p*‐value of the fixed‐effects model = 0.699), concluding that the effect size did not depend on the risk of bias score.

#### 3.5.2 Disease‐Free Survival

Meta‐analysis of the fixed‐effects model showed an HR of 2.07 (1.80–2.39, *p*‐value < 0.001) (Figure S2A) with a low heterogeneity between studies (*I* ^2^ = 0%). Considering subgroup meta‐analysis, regarding tumor subsite, the fixed‐effects model demonstrated comparable HRs for oral cavity and tongue, respectively, 2.10 (1.74–2.53, *I* ^2^ = 0%) and 1.95 (1.53–2.49, *I* ^2^ = 0%). There was only one study conducted on nasopharynx and only one study on larynx (Table S6) (ANOVA‐Q test *p*‐value = 0.340). Likewise, according to staging, an HR of 2.12 (1.78–2.52, *I* ^2^ = 0%) was found with the fixed‐effects model considering the studies that considered any stage of the disease, and a similar HR of 1.98 (1.54–2.55, *I* ^2^ = 0%) was calculated for cT1/2‐N0 stage tumors (Table S6) (ANOVA‐Q test *p*‐value = 0.674). Similarly, for the continents, the fixed‐effects model showed similar HRs for Asia, Europe, and America, respectively, 2.16 (1.78–2.62, *I* ^2^ = 0%), 1.87 (1.47–2.39, *I* ^2^ = 10.85%), and 2.33 (1.51–3.60, *I* ^2^ = 0%) (Table S6) (ANOVA‐Q test *p*‐value = 0.570). Meta‐regression by year of publication did not find any statistically significant correlation (*p*‐value of the fixed‐effects model = 0.444), concluding that the effect size did not depend on the year of publication (Figure S6). Leave‐one‐out method showed a strong consistency in (Figure S7). The presence of low publication bias was highlighted by the trim and fill method, which found two trimmed studies graphically represented by the funnel plot. However, Egger's linear regression test showed the absence of publication bias (pEgger = 0.111). Trimmed studies yielded a new HR of 2.01 (1.75–2.31; *p*‐value < 0.001) with a funnel plot with a skewness to the right (Figure S2B). Regarding RoB assessment, meta‐regression showed no statistically significant difference (*p*‐value of the fixed‐effects model = 0.727), concluding that the effect size did not depend on the risk of bias score.

**FIGURE 2:**
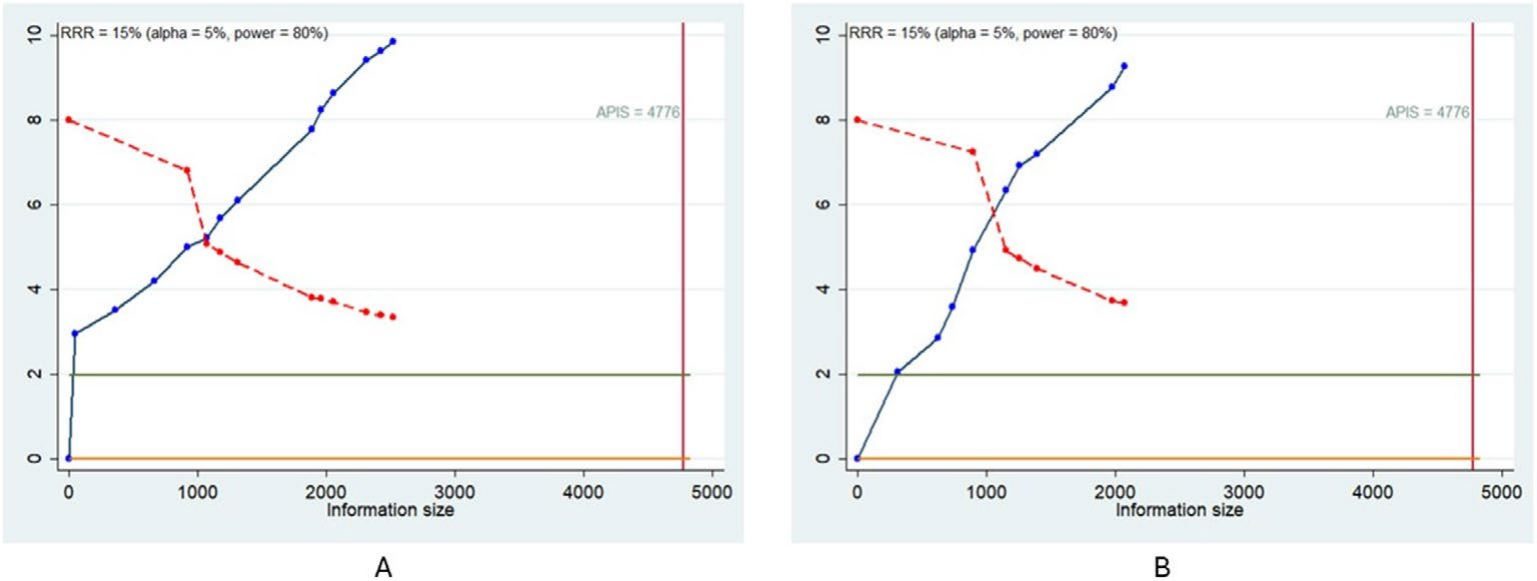
Trial sequential analysis related to the association between Tumor Stroma Ratio and disease‐specific survival (DSS) (A), and disease‐free survival (DFS) (B). APIS, light green line (*Z* = 1.98); dashed red line (monitoring boundary); blue line (cumulative z curve); red line (sample size).

TSA supports the statistical significance of the meta‐analytic results as the cumulative z‐curve (blue line) intersected the monitoring boundary (dotted red line), but this did not reach the required sample size (APIS, vertical red line—4776 patients). These results suggest that, although the pooled effect is statistically significant, regarding sample size, the result is not definitive, and future studies are necessary to be conclusive (Figure 2A).

#### 3.5.3 Disease‐Specific Survival

**FIGURE 3:**
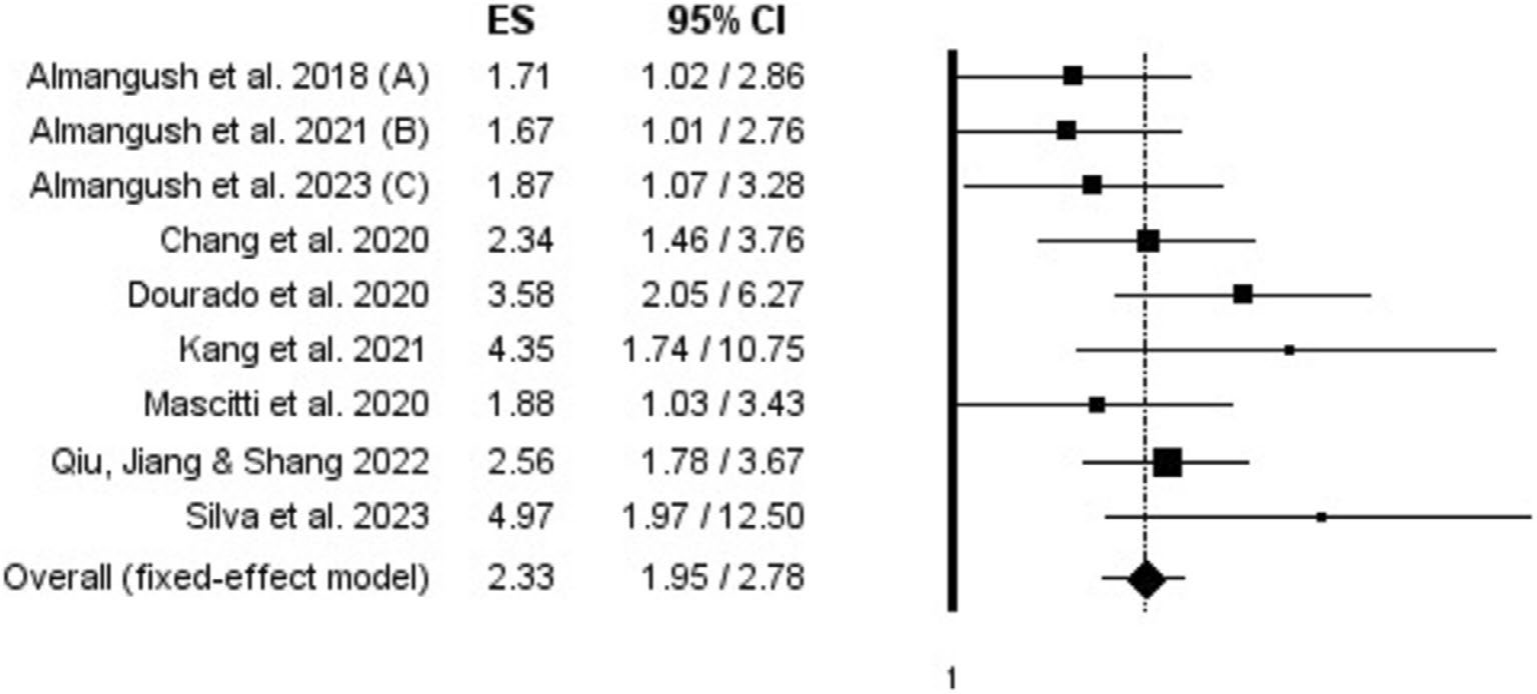
Forest plots of the association of tumor stroma ratio with disease‐specific survival (DSS) (fixed‐effects model).

Meta‐analysis of the fixed‐effects model showed an HR of 2.33 (1.95–2.78, *p*‐value < 0.001) (Figure 3). Heterogeneity was low between studies (*I* ^2^ = 27.72%). In the subgroups meta‐analysis, concerning tumor subsite, the fixed‐effects model demonstrated an HR of 2.67 (2.07–3.5, *I* ^2^ = 0%) in the oral cavity studies and an HR of 1.91 (1.43–2.56; *I* ^2^ = 16.50%) when studies considered exclusively tongue. One study considered nasopharynx (Table S7) (ANOVA‐Q test *p*‐value = 0.185). When considering any stage tumors, the fixed‐effects model showed an HR of 2.50 (2.02–3.09, *I* ^2^ = 0%), and an HR of 1.69 (1.18–2.42, *I* ^2^ = 0%) when only cT1/2‐N0 stages tumors were considered (Table S7) (ANOVA‐Q test *p*‐value = 0.066). Accounting for continent groups, the fixed‐effects model demonstrated an HR of 3.91 (2.42–6.31, *I* ^2^ = 0%) in America, an HR of 2.61 (1.98–3.43, *I* ^2^ = 0%) in Asia, and an HR of 1.77 (1.35–2.32, *I* ^2^ = 0%) in Europe (Table S7) (ANOVA‐Q test *p*‐value = 0.01). Meta‐regression by year of publication observed no statistically significant correlation (*p*‐value of the fixed‐effects model = 0.484), achieving that the effect size did not depend on the year of publication (Figure S8). Leave‐one‐out method supported our results as in (Figure S9). The presence of publication bias was highlighted by the trim and fill method, which found one new unpublished “study” through the fixed‐effects model, fully illustrated by the funnel plot. On the other hand, Egger's linear regression test showed the absence of publication bias (pEgger = 0.272) with an estimated HR of 2.26 (1.90–2.69; *p*‐value < 0.001) (Figure S3). Regarding RoB assessment, meta‐regression showed no statistically significant difference (*p*‐value of the fixed‐effects model = 0.515), concluding that the effect size did not depend on the risk of bias score.

The statistical significance of the meta‐analytic finding was confirmed by TSA since the cumulative z‐curve (blue line) crossed the monitoring boundary (dotted red line), but this did not reach the required sample size (APIS, vertical red line—4776 patients), and other studies are necessary to define this data as conclusive (Figure 2B).

#### 3.5.4 Clinical Pathological Analysis

Data were grouped by the Mantel–Haenszel method to obtain a cumulative OR and respective 95% CI on each clinical‐pathological aspect related to TSR. Patients with low TSR was positively associated with LNM development (OR = 1.76; 95% CI 1.15–2.70, *p* = 0.01; Figure S10A), staging III‐IV (OR = 1.48; 95% CI 1.21–1.82, *p* = 0.0002; Figure S10B) and T stage 3–4 (OR = 1.49; 95% CI 1.14–1.94, *p* = 0.004; Figure S10C). Additionally, TSR was not associated with either grading 2–3 (OR = 1.08; 95% CI 0.81–1.43, *p* = 0.62; Figure S10D) or sex (OR = 0.97; 95% CI 0.80–1.17, *p* = 0.62; Figure S10E).

## 4 Discussion

Interest in cancer biomarkers, especially prognostic ones, has intensified in recent years due to their potential for patients' stratification risk and personalized treatment strategies in HNSCC (Gleber‐Netto et al. 2015). The findings from our systematic review strongly indicate that low TSR in HNSCC correlates with poorer OS, DFS, and DSS, as well as with advanced clinical stage, higher T status, and presence of LNM. In this vein, the stromal component of HNSCC, and more generally the tumor microenvironment (TME), has been established as a critical factor influencing tumor development (Peltanova et al. 2019; Anderson and Simon 2020).

Generally, TSR reflects the proportion of stromal to tumor tissue, with high stromal content often linked to worse outcomes, and thus not only captures the structural balance within the TME but also integrates functional insights, such as immune activity and stromal contributions.

Initially conceptualized in Stephen Paget's 1889 “seed and soil theory,” the TME is now recognized as a critical factor in cancer progression (Paget 1989). It comprises cancer cells, immune cells, cancer‐associated fibroblasts (CAFs), endothelial cells, extracellular matrix (ECM), and soluble factors, all interacting dynamically to support tumor growth, invasion, and metastasis.

CAFs are key players within the TME, driving cancer progression through ECM remodeling, cytokine secretion, and angiogenesis. By producing and modifying ECM components like collagen and fibronectin, CAFs increase tissue stiffness and form a desmoplastic stroma that supports cancer cell survival and migration. They also secrete pro‐tumorigenic factors such as TGF‐β, IL‐6, and VEGF, which enhance proliferation, angiogenesis, and immune evasion (Graizel et al. 2020). The heterogeneity of CAFs contributes further to their influence, with subpopulations such as inflammatory CAFs promoting metastasis and chemoresistance, while myofibroblast‐like CAFs enhance invasion and create physical barriers to therapies (Yang et al. 2023). The ECM itself is a crucial component of the TME, facilitating tumor cell invasion and metastasis. Matrix metalloproteinases (MMPs), particularly MT1‐MMP, degrade ECM elements like collagen, laminin, and fibronectin, enabling cancer cell detachment and migration. MMPs also promote epithelial‐to‐mesenchymal transition, which enhances tumor invasiveness by downregulating epithelial markers like E‐cadherin and upregulating mesenchymal traits (Kessenbrock et al. 2010; Mustafa et al. 2022; Lorenzo‐Pouso et al. 2023).

Immune cells within the TME can employ both antitumorigenic and pro‐tumorigenic effects, depending on the context. Cytotoxic T (CD8+) and T‐helper 1 (Th1) cells, through the secretion of cytokines like interferon‐gamma (IFN‐γ), are associated with positive prognostic outcomes in many cancers, including HNSCC. Conversely, regulatory T cells (Tregs) and tumor‐associated macrophages (TAMs), particularly the M2 phenotype, often contribute to an immunosuppressive environment that supports tumor growth and metastasis (El‐Kenawi et al. 2020; Anderson and Simon 2020). Tumor‐infiltrating lymphocytes (TILs), particularly CD8+ and CD4+ T cells, have emerged as crucial prognostic biomarkers in HNSCC (Troiano et al. 2020) with a recent meta‐analysis by Torri et al. (2024) indicating that a high TIL count is associated with improved survival outcomes.

Therefore, the stromal component plays a critical role in identifying prognostic markers in tumors, with most studies, including those on the TSR, relying on H&E‐stained sections (Ekanayaka and Tilakaratne 2024). However, research on bulk tissues faces significant limitations in distinguishing the contributions of TME components, such as immune and stromal cells, to cancer progression. Advances in the field of single‐cell and spatial multiomics technologies offer valuable insights into the interactions among diverse cell types within tumor tissues (Kim et al. 2020; Vandereyken et al. 2023). In this context, spatial transcriptomics emerges as a promising technology that maps gene expression within tumor and stromal regions, overcoming the molecular limitations of H&E‐based TSR evaluation (Jiang et al. 2024). This approach enables a more detailed characterization of key TME components, including CAFs, immune cells, and extracellular matrix signatures, thus deepening our understanding of tumor‐stroma interactions and paving the way for the development of more robust and reliable prognostic models in the future (Choi et al. 2023; Dai et al. 2024). Nevertheless, the majority of studies to date still rely on H&E‐based evaluation, as this methodology remains the most cost‐effective, simple to perform, and rapid approach, making it better suited to routine clinical practice.

Regarding TSR, previous meta‐analyses have examined the association with HNSCC. However, they did not specifically investigate its prognostic significance across subgroups, resulting in inconclusive findings (Dolens et al. 2021; Almangush et al. 2021a; Morais et al. 2022). Given the diverse biological mechanisms and prognostic implications observed among different subtypes of the head and neck, it is imperative to conduct separate analyses to achieve a better understanding (Caponio et al. 2020) and improve classification (Perez Sayans et al. 2019). Furthermore, it should be noted that the search strategy and inclusion criteria employed in this study enabled the inclusion of a higher number of studies and patients than previous studies, thereby rendering these results, in our view, more robust.

To evaluate the quality of the included studies, we exploited the REMARKS tool, revealing disparities in rigor across studies. The “statistics” section underscored significant biases in several studies due to selective reporting of survival parameters. Moreover, the findings were partially limited by a lack of stringent multivariate analyses (Almangush et al. 2017). Another notable issue surfaced in the “Classical Prognostic Factors” section, where studies frequently lacked clear or reliable methodologies, compounded by inconsistencies in reporting “samples” and “clinical data” in primary literature.

A notable strength in the most studies was the dichotomization of patients into two groups, namely stroma‐rich (high stroma, low tumor cells, low TSR < 50%) and stroma‐poor (low stroma, high tumor cells, high TSR ≥ 50%) and the adoption of this specific cut‐off is beginning to emerge as a potential “new gold standard”. However, only a few studies reported inter‐rate or intra‐rate agreement, with data available in just six of the included reports. Cohen's kappa coefficient values ranged from 0.85 to 0.96, indicating good to great interobserver agreement and strong reproducibility where assessed, as shown in Table 1. As previously mentioned, the technique for measuring TSR lacks full standardization, resulting in variability in evaluation methods, including visual and digital approaches, scoring protocols, and the tumor areas assessment. In this systematic review, both visual and digital methods were employed, while predominantly following the protocols proposed by Van Pelt et al. for colorectal carcinoma, or minimal variations (Wright et al. 2021; van Pelt et al. 2018). Furthermore, most of the studies evaluated TSR at the invasive front of the tumor, with pathologists using various tumor regions to ensure measurement accuracy (Morais et al. 2022).

Despite these methodological differences, results across studies tend to be consistent. Conditioning on a collider generally introduces selection bias in causal analysis. Similarly, conditioning on a prognostic factor that is independent of the exposure can also result in selection bias if the exposure has a non‐null effect on the outcome and the association between the noncollider and the outcome varies across levels of the exposure (Greenland 1977). In the context of our pooled analyses, this highlights the complexity of integrating TSR with other prognostic markers (de Morais et al. 2024). There is ongoing debate regarding whether TSR offers additional prognostic value when combined with established markers such as tumor differentiation grade, DOI (Almangush et al. 2020) and staging (Mascitti et al. 2020). Consequently, we advocate for future studies on TSR in HNSCC to employ clear, reproducible methods and present data that facilitate comparison with our findings.

Moreover, our meta‐analysis is subject to certain limitations. First, the subgroup analyses based on cancer type were constrained by a limited number of relevant studies and small sample sizes, rendering the consolidated outcomes less reliable. Therefore, studies with larger cohorts are imperative to establish a definitive TSR value for the prognosis of various carcinomas. From the HR, sample number, and standard error data extracted from the meta‐analysis, the TSA analysis was also conducted particularly for DFS and DSS. These results suggest that, although the pooled effects are statistically significant, regarding sample size, the result remains not totally definitive (Mariani et al. 2022). Although the TSA results indicate robust statistical power, some scholars suggest it may be premature to assert that additional studies on the topic are unnecessary (Kang 2021). This caution stems from the inherent challenge in constructing monitoring boundaries for meta‐analyses, unlike the controlled environment of individual randomized clinical trials where stopping rules for discontinuation can be implemented to manage new evidence generation. Therefore, interpreting TSA findings should mainly focus on detecting potential false positives or false negatives within the meta‐analysis framework (i.e., type I errors and type II errors) (Wetterslev et al. 2017).

Secondly, low heterogeneity was observed, particularly concerning primary outcomes. Although not always statistically significant, differences in pooled estimations were noted across tumor subsites and geographical locations. Mechanistic explanations for these observations are currently lacking. However, it is hypothesized that exposure to specific risk factors may account for variations in tumor sites and subsites as well as geographical disparities. Information bias, referring to errors introduced during the assessment of exposure, events, or other covariates in the study population, likely played a role (Delgado‐Rodriguez and Llorca 2004). Additionally, variability in follow‐up durations across studies contributed to the observed heterogeneity to some extent (Seoane et al. 2010). Controlling these biases in epidemiological studies is challenging, particularly factors influencing selection probabilities, such as case–control status, which may impact exposure assessment (Fahey et al. 2014; Hernan 2017). Curiously, the observed results are independent of the intrinsic quality of the studies, as the magnitude of the association remains similar between studies deemed high quality and those with lower quality scores according to REMARKS. Bearing in mind all these limitations, the robust nature of the current meta‐analysis is evidenced by the forest plot, which indicates a strong association for most evaluated outcomes. This robustness is further confirmed by our sensitivity analysis.

Third, 13 of the 19 included studies lacked agreement rate metrics for TSR assessments, which significantly affects the comparability of results and weakens the overall conclusions. Reliability is a critical parameter to ensure consistency between raters and validate the robustness of data. To address this limitation, future studies should include detailed reliability metrics, such as Cohen's kappa (*κ*), along with percent agreement (*p*_o_) and chance agreement (*p*). Reporting these values would enable a more comprehensive analysis of interrater reliability in meta‐analyses, ultimately strengthening the methodological quality and enhancing the reproducibility of results in this field.

Nevertheless, while more than half of the included studies surpassed the cut‐off point ≥ 4, only a third fulfilled all six REMARK criteria, highlighting a potential limitation that should be taken into account when interpreting these findings.

## 5 Conclusions

In conclusion, mapping TSR offers crucial insights into long‐term survival among patients affected by HNSCC. Further studies investigating the predictive utility of these measurements are warranted. Establishing standardized international guidelines for TSR assessment could enhance result consistency and facilitate future clinical applicability. With increased consistency and robustness of evidence supporting these findings, the incorporation of TSR into AJCC staging for HNSCC could be considered only after its validation in well‐designed cohort studies, overcoming the limitations raised in this study. To mitigate the proliferation of small studies employing disparate methodologies, national regulatory bodies and international health organizations can play a pivotal role in coordinating and harmonizing the design of subsequent studies.

## Author Contributions

**Gennaro Musella:** conceptualization, methodology, software, data curation, formal analysis, funding acquisition, project administration, writing – original draft. **Martina Coppini:** methodology, validation, writing – original draft. **Fábio França Vieira E Silva:** software, investigation, formal analysis, visualization, writing – original draft. **Giuseppina Campisi:** data curation, visualization, writing – review and editing. **Mario Pérez‐Sayáns:** data curation, investigation, resources, writing – original draft, writing – review and editing. **Vito Carlo Alberto Caponio:** conceptualization, software, data curation, supervision, funding acquisition, project administration, resources, writing – original draft, writing – review and editing. **Alejandro I. Lorenzo‐Pouso:** conceptualization, data curation, validation, supervision, writing – original draft, writing – review and editing.

## Disclosure

The authors authorize the reproduction of material from other sources.

## Ethics Statement

The authors have nothing to report. All included studies were assumed to have followed the necessary ethical guidelines as reported by their respective authors.

## Consent

The authors have nothing to report.

## Conflicts of Interest

The authors declare no conflicts of interest.

## Supporting information


Data S1.

## Data Availability

The data supporting the findings of this study are available from the corresponding author, Prof. Vito Carlo Alberto Caponio. All relevant data are included within the article and its Supporting Information.
