# Supplementary material for: Tumor–Stroma Ratio in Head and Neck Squamous Cell Carcinoma: A Systematic Review and Meta‐Analysis
Source: Oral Dis. 2025 Mar 24;31(8):2382–93. doi: 10.1111/odi.15319 (PMC12423486; doi:10.1111/odi.15319)

**Appendix to the manuscript**

**Prognostic and Clinicopathological Significance of Tumor-Stroma Ratio in Head and Neck Squamous Cell Carcinoma: Meta-Analysis and Trial Sequential Analysis**

**Table of contents**

**Table S1.** Search strategy for each database.

**Table S2.** Evaluation methodology of risk of bias of each individual study according to an ad hoc adaptation of Reporting Recommendations for Tumor Marker Prognostic Studies (REMARK) guidelines.

**Table S3.** Main characteristics and survival analysis data of each included study.

**Table S4.** Quality assessment according to REMARKS guidelines.

**Table S5.** Subgroup analysis for overall survival (OS) stratified according to the continent of patients; tumor subsite, and tumor staging.

**Table S6.** Subgroup analysis for disease-free survival (DFS) stratified according to the continent of patients; tumor subsite, and tumor staging.

**Table S7.** Subgroup analysis for disease-specific survival (DSS) stratified according to the continent of patients; tumor subsite, and tumor staging.

**Fig. S1**. A) Forest plots of the association of tumor stroma ratio with overall survival (OS) (fixed-effects model). B) A funnel plot of estimated logHR against its standard error, graphically representing the analysis of effects on OS. The black circles represent the studies published and the black bubble represents the non-published studies estimated by the trim-and-fill method.

**Fig. S2**. A) Forest plots of the association of tumor stroma ratio with disease-free survival (DFS) (fixed-effects model). B) A funnel plot of estimated logHR against its standard error, graphically representing the analysis of effects on DFS. The black circles represent the studies published and the black bubble represents the non-published studies estimated by the trim-and-fill method.

**Fig. S3**. A funnel plot of estimated logHR against its standard error, graphically representing the analysis of effects on DSS. The black circles represent the studies published and the black bubble represents the non-published studies estimated by the trim-and-fill method.

**Figure S4.** Bubble plot graphically representing the potential effect of year of publication (n) on overall survival.

**Figure S5.** Leave-one-out analysis for overall survival.

**Figure S6.** Bubble plot graphically representing the potential effect of year of publication (n) on disease-free survival.

**Figure S7.** Leave-one-out analysis for disease-free survival.

**Figure S8.** Bubble plot graphically representing the potential effect of year of publication (n) on disease-specific survival.

**Figure S9.** Leave-one-out analysis for disease-specific survival.

**Fig. S10** Meta-analysis comparing clinical-pathological aspects with the tumor-stroma ratio in included studies. (A) Meta-analysis related to LNM *p = 0.01; (B) Meta-analysis related to staging III-IV *p = 0.0002; (C) Meta-analysis related to T status 3–4 *p = 0.004; (D) Meta-analysis related to Grading 2–3 *p = 0.62; (E) Meta-analysis related to male sex*p = 0.62.

**Table S1.** Search strategy for each database

| **Database** | **Search strategy** |
| --- | --- |
| **PubMed** | ("Tumour-Stroma Ratio" OR “Tumor-Stroma Ratio” OR “TSR”) AND (“Mouth Neoplasms”[MeSH] OR “oral cancer” OR “oral squamous cell carcinoma” OR “OSCC” OR "Head and Neck Neoplasms"[Mesh] OR “head and neck squamous cell carcinoma” OR HNSCC OR “oropharynx squamous cell carcinoma” OR “OPSCC” OR “larynx cancer” OR “oropharynx cancer” OR “larynx squamous cell carcinoma” OR “hypopharynx cancer” OR “hypopharynx squamous cell carcinoma”) AND prognosis |
| **Scopus** | TITLE-ABS-KEY ( ( "Tumour-Stroma Ratio" OR "histological parameter" OR "Tumor-Stroma Ratio" OR "TSR" ) AND ( "oral cancer" OR "oral squamous cell carcinoma" OR "OSCC" OR "head and neck squamous cell carcinoma" OR hnscc OR "oropharynx squamous cell carcinoma" OR "OPSCC" OR "larynx cancer" OR "oropharynx cancer" OR "larynx squamous cell carcinoma" OR "hypopharynx cancer" OR "hypopharynx squamous cell carcinoma" ) ) |
| **Web of Science** | ( "Tumour-Stroma Ratio" OR "histological parameter" OR "Tumor-Stroma Ratio" OR "TSR" ) AND ( "oral cancer" OR "oral squamous cell carcinoma" OR "OSCC" OR "head and neck squamous cell carcinoma" OR hnscc OR "oropharynx squamous cell carcinoma" OR "OPSCC" OR "larynx cancer" OR "oropharynx cancer" OR "larynx squamous cell carcinoma" OR "hypopharynx cancer" OR "hypopharynx squamous cell carcinoma" ) (Topic) |
| **EMBASE** | ('tumour-stroma ratio' OR 'tumor stroma ratio'/exp OR 'tumor stroma ratio' OR 'tumour stroma ratio' OR 'histological parameter' OR 'tumor-stroma ratio' OR 'tsr') AND ('mouth tumor'/exp OR 'mouth tumor' OR 'oral cancer'/exp OR 'oral cancer' OR 'oral squamous cell carcinoma'/exp OR 'oral squamous cell carcinoma' OR 'oscc' OR 'head and neck neoplasms'/exp OR 'head and neck neoplasms' OR 'head and neck squamous cell carcinoma'/exp OR 'head and neck squamous cell carcinoma' OR 'hnscc'/exp OR 'hnscc' OR 'oropharynx squamous cell carcinoma'/exp OR 'oropharynx squamous cell carcinoma' OR 'opscc' OR 'larynx cancer'/exp OR 'larynx cancer' OR 'oropharynx cancer'/exp OR 'oropharynx cancer' OR 'larynx squamous cell carcinoma'/exp OR 'larynx squamous cell carcinoma' OR 'hypopharynx cancer'/exp OR 'hypopharynx cancer' OR 'hypopharynx squamous cell carcinoma'/exp OR 'hypopharynx squamous cell carcinoma') |

**Table S2.** Evaluation methodology of risk of bias of each individual study according to an ad hoc adaptation of Reporting Recommendations for Tumor Marker Prognostic Studies (REMARK) guidelines.

| **Parameters** | **Criteria** |
| --- | --- |
| 1. Sample size | The sample size was deemed inadequate if it included fewer than 90 patients, determined through comparison and an assessment of the statistical significance that the sample could produce. |
| 2. Clinical data from the cohort | Clinical information was regarded as inadequate when it was either not provided or when the study failed to detail standard conditions that could affect the prognosis and did not directly compare clinical variables with the tumor stroma ratio. |
| 3. Histopathology/ Immunohistochemistry | The histopathology/immunohistochemistry analysis was deemed inadequate due to documented technical failures or when the evaluation criteria and the cut-off for the tumor stroma ratio were not provided or deemed disproportionate. |
| 4. Prognosis | The prognostication was considered inadequate if the follow-up was not informed, or when the follow-up time was not a standard for an accurate and reliable prognostic evaluation. |
| 5. Statistics | The statistical parameter was deemed inadequate if the analyses were considered inappropriate, the sample size was insufficient, the hazard ratios (HRs) were not provided in numerical values, or if the HRs or their 95% confidence intervals (CIs) were considered abnormal. |
| 6. Conventional prognostic factors | The classical prognostic factors were considered inadequate when the study was limited prognostic analysis, or when it did not clearly and specifically address the numbers referring to the classical prognostic factors. |

**Table S3.** Main characteristics and survival analysis data of each included study.

| **Authors** | **Country** | **Year** | **Subsite** | **Sample Size** | **Cut-Off** | **Magnification** | **Staining** | **Time of Follow-Up (months)** | **Staging Edition** | **Stage included** | **Survival Analysis** |
| --- | --- | --- | --- | --- | --- | --- | --- | --- | --- | --- | --- |
|  |  |  |  |  |  |  |  |  |  |  |  |
|  |  |  |  |  |  |  |  |  |  |  |  |
| Alessandrini et al. | Italy | 2024 | Larynx | 43 | 50% | 100x | H&E | 76,2 ± 46,5 [mean] | 8th AJCC | ANY | DFS |
| Almangush et al. (A) | Finland, Brazil | 2018 | Tongue | 311 | 50% | 100x | H&E | N/A | 7th AJCC | cT1/2N0 | DFS, DSS |
| Almangush et al. (B) | Brazil | 2021 | Tongue | 308 | 50% | 100x | H&E | N/A | not specified | cT1/2N1 | DFS, DSS, OS |
| Almangush et al. (C) | Finland | 2023 | Nasopharynx | 115 | 50% | 100x | H&E | N/A | 7th UICC | ANY | OS, DSS |
| Chang et al. | Taiwan | 2024 | Oral cavity | 162 | 50% | 100x | H&E | N/A | 8th AJCC | ANY | OS, DSS |
| Dourado et al. | Brazil | 2020 | Oral cavity | 254 | 50% | 100x | H&E | 47 (range 1-178) [mean] | 7th TNM (not specified) | ANY | DFS, DSS |
| Huang et al. | China | 2022 | Oral cavity | 151 | 50% | 100x | H&E | 44,2 (range 3,2–83,0) [mean] | 7th AJCC | cT1/2N0 | DFS, OS |
| Kang et al. | China | 2021 | Tongue | 103 | 50% | 100x | H&E | 85 [median] | 8th AJCC | ANY | DFS, DSS |
| Karpathiou et al. | French | 2019 | Head&Neck | 266 | 50% | 100x | H&E | N/A | 7th UICC | ANY | OS |
| Knief et al. | Germany | 2024 | Oral cavity | 107 | 50% | 100x | H&E | 57,7 (range 1–215) [mean] | 8th TNM (not specified) | ANY | OS, DFS |
| Mascitti et al. | Italy | 2020 | Tongue | 211 | 50% | 200x | H&E | 50.4 ± 36.8 (range 3– 120) [mean] | 7th AJCC | ANY | DFS, DSS, OS |
|  |  |  |  | 139 | 50% | 200x | H&E |  | 8th AJCC | ANY | DFS, DSS, OS |
| Niranjan et al. | India | 2018 | Oral cavity | 60 | 50% | 100x | H&E | 36 (range 14-48) [mean] | not specified | ANY | DFS, OS |
| Qiu, Jiang & Shang | China | 2022 | Oral cavity | 581 | 50% | 100x | H&E | 30.33 (range 1–36) [mean] | not specified | ANY | DFS, DSS |
| Sakai et al. | Japan | 2022 | Tongue | 70 | 50% | 200x | H&E | 47 (range 5–125) [median] | UICC/AJCC, 7th and 8th editions | cT1/2N0 | DFS, LNM |
| Silva et al. | Brazil | 2023 | Oral cavity | 95 | 50% | N/A | H&E | range 6 to 178 months | 7th UICC | ANY | DFS, DSS |
| Sung et al. | South Corea | 2020 | Oral cavity | 256 | Median [0.96 (0.53, 1.84)] | 40x | H&E | 66 (range: 20–192) [months] | 8th AJCC | ANY | DFS, OS |
| Unlu et al. | Turkey | 2013 | Larynx | 85 | 50% | 200x | H%E | 48 (range 3-194) [median] | not specified | ANY | OS |
| Wang et al. | China | 2023 | Oral cavity | 114 | 50% | 100x | H&E | 52.35 ± 20.04 (range 1–76) [mean] | 8th AJCC | ANY | OS, DFS, MFS |
| Zhang et al. | China | 2014 | Nasopharynx | 93 | 50% | 100x | H&E | 65 (range 12-108) [median] | 6th AJCC | ANY | OS, DFS |

**Table S4.** Quality assessment according to REMARKS guidelines.

*Abbreviation: IHC, Histopathology/ Immunohistochemistry.*

| **Authors** | **Country** | **Year** | **Samples** | **Clinical**  **Data** | **IHC** | **Prognostication** | **Statistics** | **Classical**  **Prognostic**  **Factors** | **Overall** |
| --- | --- | --- | --- | --- | --- | --- | --- | --- | --- |
| **Alessandrini *et al.*** | Italy | 2022 | I | A | A | A | I | I | 3 |
| **Almangush *et al.* (A)** | Finland | 2018 | I | I | A | A | A | A | 4 |
| **Almangush *et al.* (B)** | Finland | 2021 | A | I | A | A | A | A | 5 |
| **Almangush *et al.* (C)** | Finland | 2023 | A | I | A | A | A | A | 5 |
| **Chang & Lee** | Taiwan | 2023 | A | A | A | A | A | A | 6 |
| **Dourado *et al.*** | Brazil | 2020 | A | A | A | A | A | A | 6 |
| **Huang *et al.*** | China | 2021 | A | A | A | A | A | A | 6 |
| **Kang *et al.*** | China | 2021 | A | A | A | A | A | A | 6 |
| **Karpathiou *et al.*** | France | 2018 | A | I | A | A | I | A | 4 |
| **Knief *et al.*** | Germany | 2024 | A | I | A | A | A | A | 5 |
| **Mascitti *et al.*** | Italy | 2020 | A | I | A | A | A | A | 5 |
| **Niranjan & Sarathy** | India | 2018 | I | A | A | I | I | I | 2 |
| **Qiu, Jiang & Shang** | China | 2022 | A | I | A | I | I | A | 3 |
| **Sakai *et al.*** | Japan | 2022 | A | I | A | I | I | A | 3 |
| **Silva *et al.*** | Brazil | 2022 | A | A | A | A | A | A | 6 |
| **Sung *et al.*** | South Korea | 2022 | A | A | I | I | A | A | 4 |
| **Ünlü *et al.*** | Turkey | 2012 | I | A | I | A | I | I | 2 |
| **Wang *et al.*** | China | 2023 | A | I | A | I | A | A | 4 |
| **Zhang *et al.*** | China | 2014 | A | A | A | A | A | A | 6 |

**Table S5.** Subgroup analysis for overall survival (OS) stratified according to the continent of patients; tumor subsite, and tumor staging.

| Overall Survival | | | | | | | |
| --- | --- | --- | --- | --- | --- | --- | --- |
| Subgroup | Variables | Number  of  Studies | Hazard  Ratio (HR) | 95%CI | p-value  HR | Heterogeneity | ANOVA-Q  test |
| Country | Europe | 6 | 1.82 | 1.51-2.19 | <0.001 | 14.67% | = 0.103 |
|  | Asia | 6 | 2.39 | 1.83–3.13 | <0.001 | 44.95% |  |
| Subsite | Nasopharynx | 2 | 2.11 | 1.37–3.23 | 0.001 | 0% | = 0.863 |
|  | Oral cavity | 6 | 2.37 | 1.56–3.60 | <0.001 | 53.16% |  |
|  | Tongue | 2 | 1.48 | 1.11-1.97 | 0.007 | 0% |  |
|  | Larynx | 1 | 1.43 | 0.47-4.28 | - | - |  |
|  | Head & Neck | 1 | 2.33 | 1.71-3.16 | - | - |  |
| Staging | Any stage | 10 | 2.14 | 1.80–2.55 | <0.001 | 7.07% | 0.087 |
|  | cT1/2-N0 | 2 | 2.83 | 0.58-13.81 | - | - |  |

**Table S6.** Subgroup analysis for disease-free survival (DFS) stratified according to the continent of patients; tumor subsite, and tumor staging.

| Disease-Free Survival | | | | | | | |
| --- | --- | --- | --- | --- | --- | --- | --- |
| Subgroup | Variables | Number  of  Studies | Hazard  Ratio (HR) | 95%CI | p-value  HR | Heterogeneity | ANOVA-Q  test |
| Country | Europe | 5 | 1.87 | 1.47-2.39 | <0.001 | 0% | 0.570 |
|  | Asia | 8 | 2.16 | 1.78-2.62 | <0.001 | 10.85% |  |
|  | South America | 2 | 2.33 | 1.51-3.60 | <0.001 | 0% |  |
| Subsite | Nasopharynx | 1 | 1.99 | 1.02–3.62 | - | - | = 0.340 |
|  | Oral cavity | 8 | 2.10 | 1.74–2.53 | <0.001 | 0% |  |
|  | Tongue | 5 | 1.95 | 1.53-2.49 | <0.001 | 0% |  |
|  | Larynx | 1 | 6.11 | 1.83-20.38 | - | - |  |
| Staging | Any stage | 10 | 2.12 | 1.78–2.52 | <0.001 | 0% | = 0.674 |
|  | cT1/2-N0 | 5 | 1.98 | 1.54–2.55 | <0.001 | 0% |  |

**Table S7.** Subgroup analysis for disease-specific survival (DSS) stratified according to the continent of patients; tumor subsite, and tumor staging.

| Disease-Specific Survival | | | | | | | |
| --- | --- | --- | --- | --- | --- | --- | --- |
| Subgroup | Variables | Number  of  Studies | Hazard  Ratio (HR) | 95%CI | p-value  HR | Heterogeneity | ANOVA-Q  test |
| Country | Europe | 4 | 1.77 | 1.35-2.32 | <0.001 | 0% | = 0.010 |
|  | Asia | 3 | 2.61 | 1.98-3.43 | <0.001 | 0% |  |
|  | South America | 2 | 3.91 | 2.42-6.31 | <0.001 | 0% |  |
| Subsite | Nasopharynx | 1 | 1.87 | 1.07–3.28 | - | - | =0.185 |
|  | Oral cavity | 3 | 2.67 | 2.07–3.45 | <0.001 | 0% |  |
|  | Tongue | 4 | 1.91 | 1.43–2.56 | 0.001 | 16.50 % |  |
| Staging | Any stage | 6 | 2.50 | 2.02–3.09 | <0.001 | 0% | - |
|  | cT1/2-N0 | 2 | 1.69 | 1.18–2.42 | 0.004 | 0% |  |

**Fig. S1**. A) Forest plots of the association of tumor stroma ratio with overall survival (OS) (fixed-effects model). B) A funnel plot of estimated logHR against its standard error, graphically representing the analysis of effects on OS. The black circles represent the studies published and the black bubble represents the non-published studies estimated by the trim-and-fill method.

A


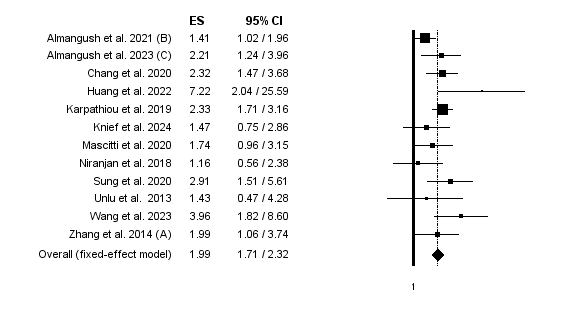


B


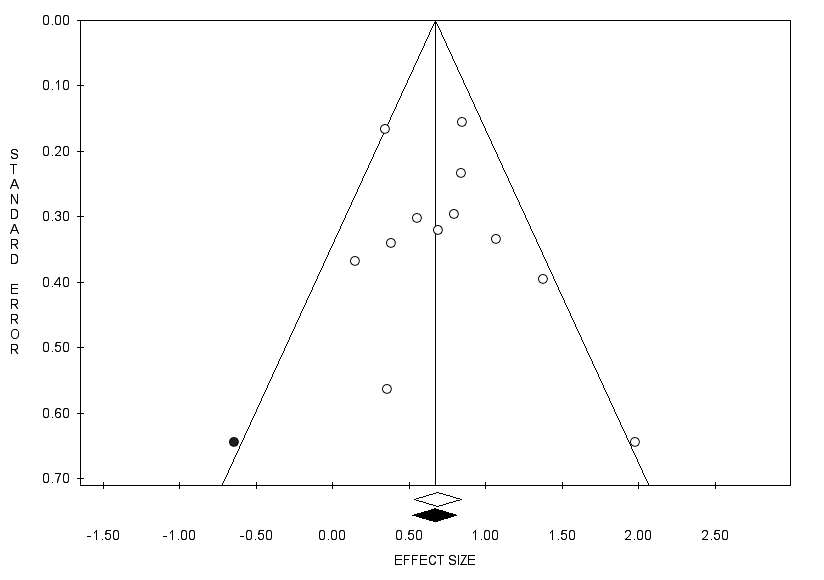


**Fig. S2**. A) Forest plots of the association of tumor stroma ratio with disease-free survival (DFS) (fixed-effects model). B) A funnel plot of estimated logHR against its standard error, graphically representing the analysis of effects on DFS. The black circles represent the studies published and the black bubble represents the non-published studies estimated by the trim-and-fill method.

A


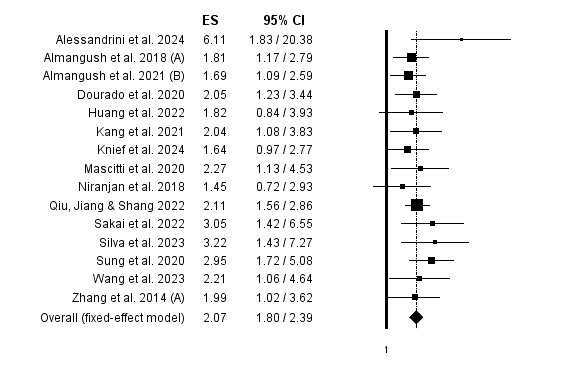


B


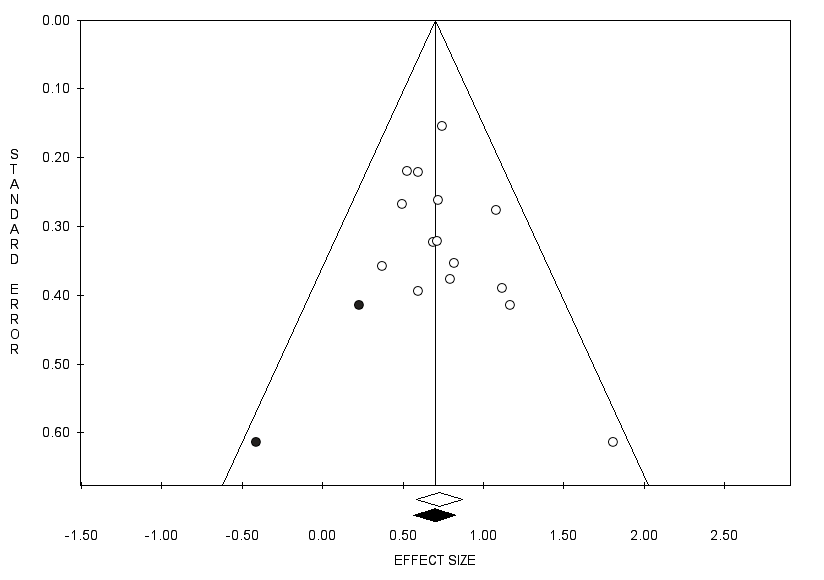


**Fig. S3**. A funnel plot of estimated logHR against its standard error, graphically representing the analysis of effects on DSS. The black circles represent the studies published and the black bubble represents the non-published studies estimated by the trim-and-fill method.


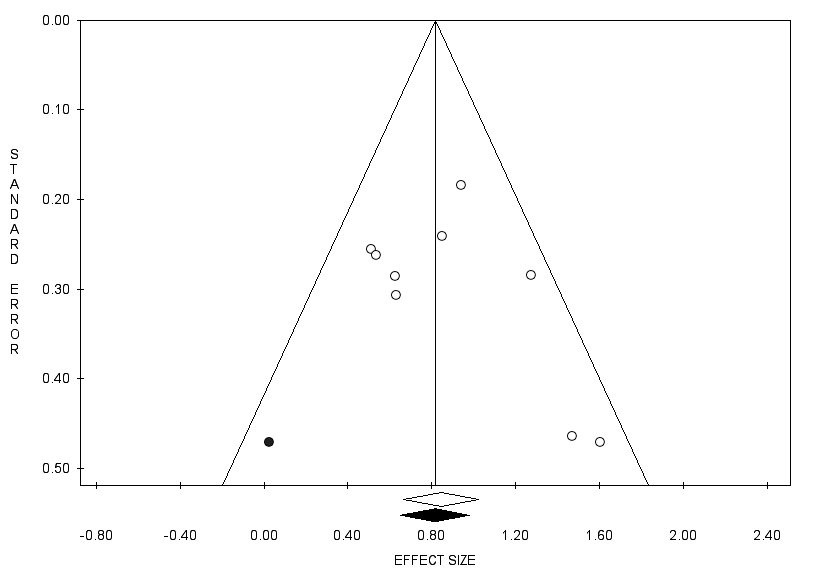


**Figure S4.** Bubble plot graphically representing the potential effect of year of publication (n) on overall survival. [The black line exhibits the fitted regression line together with circles representing the estimates from each individual study, sized according to the precision of each estimate (the inverse of its within-study variance)].


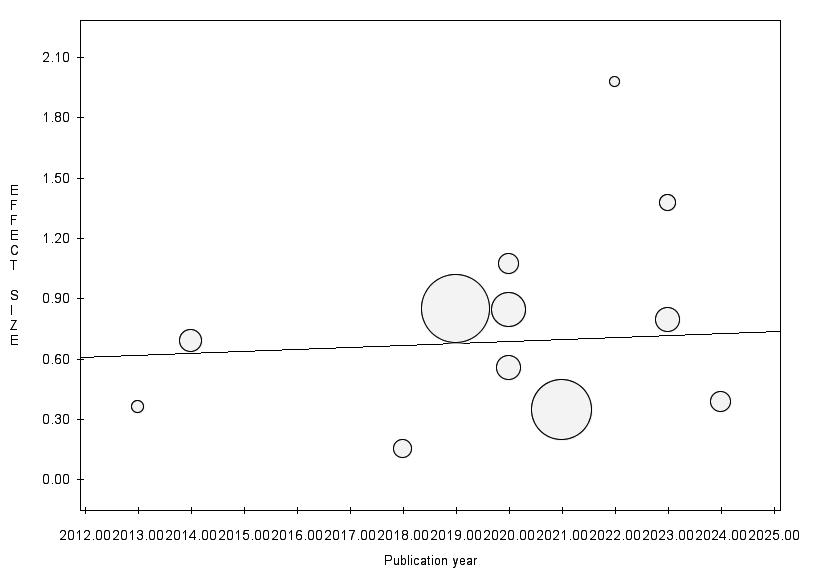


**Figure S5.** Leave-one-out analysis for overall survival. [Sensitivity analysis (“leave-one-out” method) of the meta-analysis results, sequentially omitting one study at a time]

**
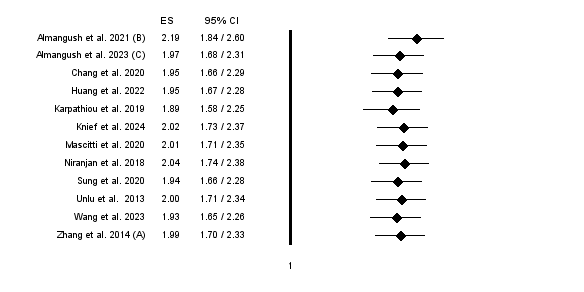
**

**Figure S6.** Bubble plot graphically representing the potential effect of year of publication (n) on disease-free survival. [The black line exhibits the fitted regression line together with circles representing the estimates from each individual study, sized according to the precision of each estimate (the inverse of its within-study variance)].


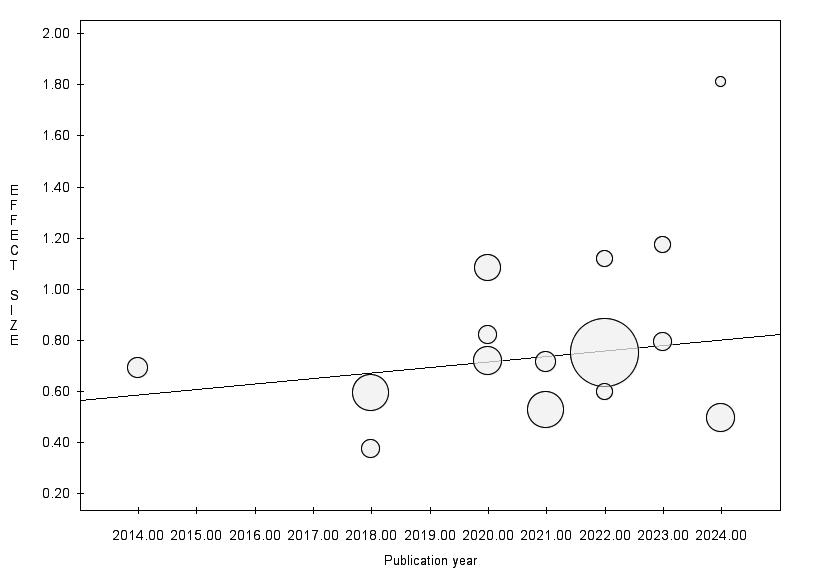


**Figure S7.** Leave-one-out analysis for disease-free survival. [Sensitivity analysis (“leave-one-out” method) of the meta-analysis results, sequentially omitting one study at a time]


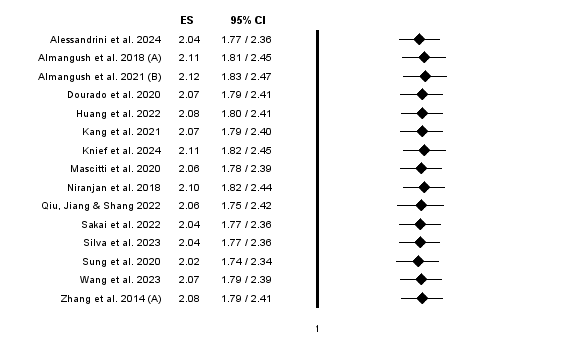


**Figure S8.** Bubble plot graphically representing the potential effect of year of publication (n) on disease-specific survival. [The black line exhibits the fitted regression line together with circles representing the estimates from each individual study, sized according to the precision of each estimate (the inverse of its within-study variance)]


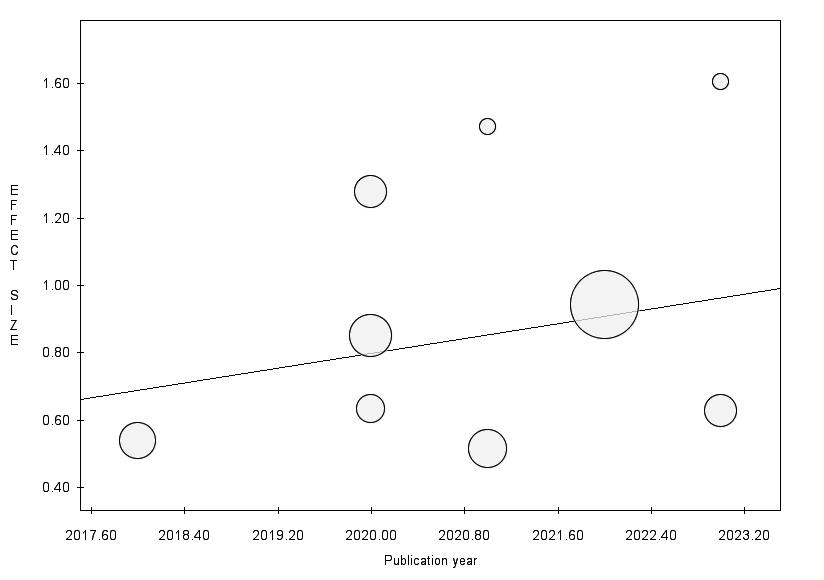


**Figure S9.** Leave-one-out analysis for disease-specific survival. [Sensitivity analysis (“leave-one-out” method) of the meta-analysis results, sequentially omitting one study at a time]


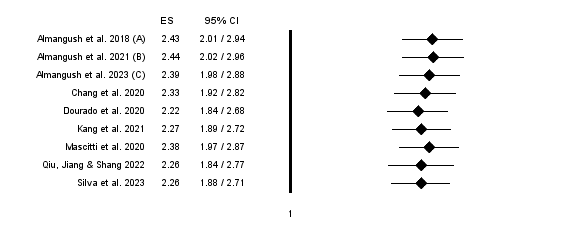


**Fig. S10** Meta-analysis comparing clinical-pathological aspects with the tumor-stroma ratio in included studies. (A) Meta-analysis related to LNM *p = 0.01; (B) Meta-analysis related to staging III-IV *p = 0.0002; (C) Meta-analysis related to T status 3–4 *p = 0.004; (D) Meta-analysis related to Grading 2–3 *p = 0.62; (E) Meta-analysis related to male sex*p = 0.62.

A


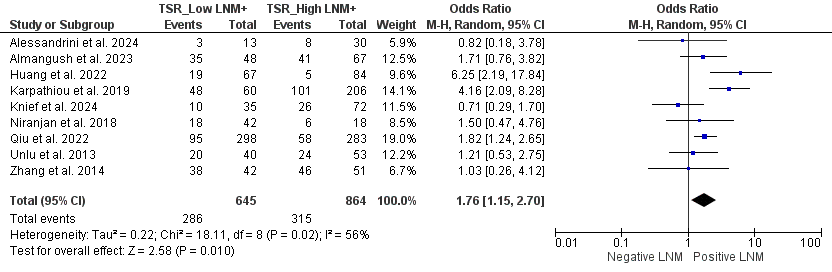


B


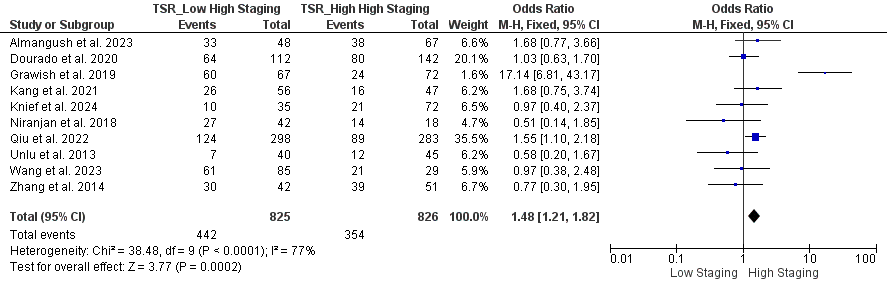


C


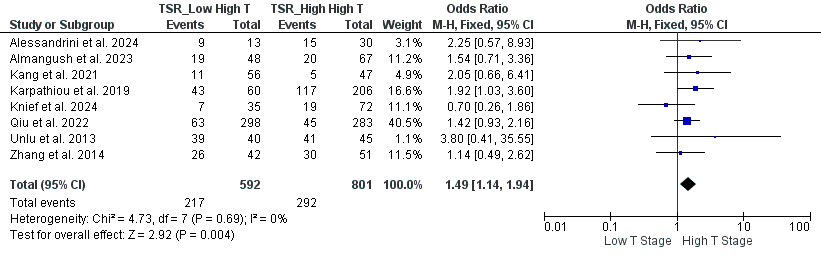


D


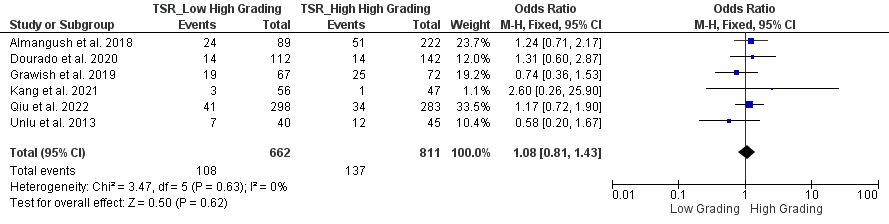


E


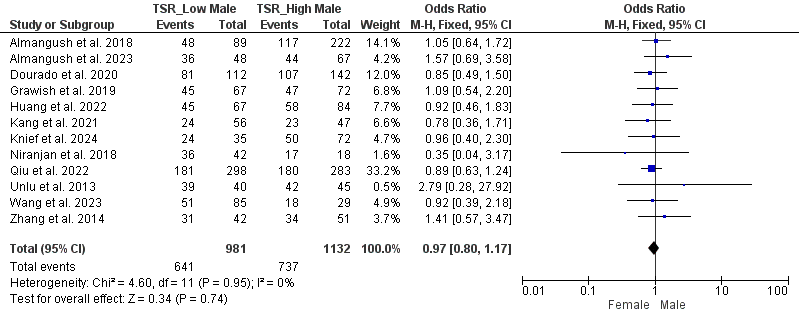

Supplement: Supplementary file 1 — Data S1. [file ODI-31-2382-s001.docx]
